# The Clinical and Surgical Characteristics of Parotid Tumors with Parapharyngeal Space Involvement—A Multicenter Experience of the Polish Salivary Network

**DOI:** 10.3390/jcm13154574

**Published:** 2024-08-05

**Authors:** Dominik Stodulski, Bogusław Mikaszewski, Paweł Witkowski, Ewelina Bartkowiak, Wioletta Pietruszewska, Izabela Olejniczak, Jarosław Markowski, Aleksandra Piernicka-Dybich, Paweł Burduk, Małgorzata Wierzchowska, Joanna Czech, Katarzyna Radomska, Alicja Chańko, Daniel Majszyk, Antoni Bruzgielewicz, Patrycja Gazińska, Małgorzata Wierzbicka

**Affiliations:** 1 Department of Otolaryngology, Faculty of Medicine, Medical University of Gdansk, 80-210 Gdansk, Poland; 2 Department of Otolaryngology and Laryngological Oncology, Poznan University of Medical Sciences, 61-701 Poznan, Poland; 3 Department of Otolaryngology, Head Neck Oncology, Medical University of Lodz, 90-419 Lodz, Poland; 4 Department of Laryngology, Faculty of Medical Sciences in Katowice, Medical University of Silesia in Katowice, 40-055 Katowice, Poland; 5 Department of Otolaryngology, Phoniatrics and Audiology, Collegium Medicum, Nicolaus Copernicus University, 87-100 Bydgoszcz, Poland; 6 Department of Otolaryngology, Pomeranian University of Medicine, 70-204 Szczecin, Poland; 7 Department of Otorhinolaryngology Head and Neck Surgery, Medical University of Warsaw, 02-091 Warsaw, Poland; 8 Biobank Research Group, Lukasiewicz Research Network—PORT Polish Center for Technology Development, 54-066 Wroclaw, Poland; 9 Research Oncology, Division of Cancer Studies, Faculty of Life Sciences and Medicine, King’s College London, London SE1 9RT, UK; 10 Department of Otolaryngology, Regional Specialist Hospital Wroclaw, Research & Development Centre, 51-124 Wroclaw, Poland; 11 Faculty of Medicine, Wroclaw University of Science and Technology, 50-370 Wroclaw, Poland; 12 Institute of Human Genetics, Polish Academy of Sciences, 01-447 Poznan, Poland

**Keywords:** parapharyngeal space, prestyloid compartment, parotid gland, tumors, salivary gland, surgery, parotidectomy

## Abstract

**Backgrounds/Objectives:** Parotid gland tumors (PGTs) with parapharyngeal space (PPS) involvement have a specific clinical course and they can be a great challenge for surgeons, especially due to more difficult approaches and the risk of serious complications. The aim of this study is to present the characteristics of PGTs with PPS involvement. **Methods**: Retrospective, multicenter analysis of 1954 primary PGTs from 5 years (2017–2021) was performed. Comparative analysis was performed between groups with and without PPS involvement and included the following clinical and histopathological data: age, sex, place of residence, tumor size, FNAC result, percentage of malignant tumors, histological diagnosis, radicality of resection, and postoperative facial nerve (FN) dysfunction. **Results:** PPS involvement was found in 114 patients (5.83%). Secondary tumors affecting the deep lobe or the entire gland were predominant (46 and 60 cases, respectively). In a univariate analysis of tumors with and without PPS involvement, statistically significant differences were found in their size > 4 cm (12.97% vs. 37.72%), percentage of malignant tumors (7.12% vs. 17.55%), incidence of Warthin Tumors (WTs) (43.58% vs. 24.56%), percentage of R1 resection (5.53% vs. 12.50%), and rate of FN paresis (17.15% vs. 53.34%). Multivariate analysis showed that tumors with PPS involvement were statistically significantly characterized by larger size (tumors > 4 cm were 2.9 times more frequent), 2 times less frequent occurrence of WTs, and 1.6 times higher risk of FN paresis. **Conclusion:** PGTs with PPS involvement show certain clinical and histological differences and require more complex surgical accesses. Therefore, they cannot be treated as "ordinary" tumors occupying the deep lobe.

## 1. Introduction

**Figure 1:**
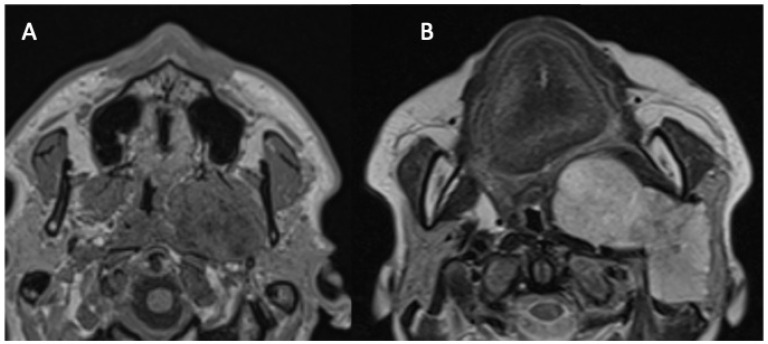
Two forms of the parapharyngeal space involvement of parotid tumors. Magnetic resonance images: axial T1WI images at the level of the hard palate. (**A**) Non-contrast image presenting an isolated PPS tumor developed from the medial protuberance of the parotid gland deep lobe. (**B**) Contrast-enhanced image showing a deep lobe tumor with the extensive involvement of the PPS.

The parapharyngeal space (PPS) has the shape of an inverted pyramid, extending from the base of the skull (temporal bone) to the hyoid bone, bounded from behind by the vertebral column and prevertebral muscles, from the adventitia by the buccopharyngeal fascia, and from the side by the condyle of the mandible and medial pterygoid muscle. The styloid process divides it into two compartments: anterior-prestyloid and posterior-poststyloid (carotid space) [1]. The vast majority of tumors developing in the prestyloid space originate in the salivary glands [1,2,3]. PPS involvement of parotid tumors has two forms. In the first (Figure 1A), the neoplasm develops in the PPS from the medial protuberance of the deep lobe in an isolated manner, but in continuity with the deep lobe, while in the second, the tumor of the deep part (the so-called deep lobe) or the entire gland grows medially through the stylomandibular tunnel (Figure 1B). 

There are also isolated salivary gland tumors growing without communication with the deep lobe, which originate not from the parotid gland but most likely from ectopic tissue or the minor salivary glands. In many publications, tumors of the deep lobe of the parotid gland are presented alongside PPS tumors. Riffat et al. consider the involvement of at least the retromandibular part of the deep lobe as a criterion for PPS involvement; however, according to the aforementioned anatomical description, "true" PSS tumors are located medial to the stylomandibular plane (stylomandibular tunnel) [1,4]. The European Salivary Gland Society (ESGS) classification of parotidectomy distinguishes five levels within the parotid gland: I and II are the upper and lower superficial lobes, III and IV are the lower and upper deep lobes, and V is the accessory gland. However, it does not include tumors with involvement of the parapharyngeal space [5]. Immediately after the publication of this classification, Fakhry, in a letter to the editors, advocated for the introduction of an additional level for the PPS in the ESGS classification; however, this proposal was ultimately not included [6,7]. 

The aim of our study was to verify a hypothesis that parotid gland tumors with parapharyngeal space extension significantly differ in clinical behavior, histology, and treatment results from those without its involvement.

## 2. Material and Methods

This study was approved by the Independent Bioethics Committee for Scientific Research (resolution nr KB—666/23) and was conducted according to the Declaration of Helsinki on biomedical research involving human subjects. Retrospective analysis of 1954 primary parotid tumors from 5 years (2017–2021) included cases recorded in the Polish Salivary Network Database by 7 university centers. 

A comparative analysis of the following clinical and histopathological data was performed between groups with and without PPS involvement: age, sex, place of residence, tumor size, fine needle aspiration cytology (FNAC) result, percentage of malignant tumors, most common histopathological diagnosis, radicality of resection, and postoperative facial nerve dysfunction. Preoperative imaging was performed in all patients (ultrasound, US, and/or magnetic resonance imaging, MRI—599, and/or contrast-enhanced computed tomography, CECT—298). A US was preformed in all cases but in isolation only in the cases with superficial (ESGS I and/or II levels) lesions.

The criterion for involvement of the PPS was based on the location or extension of a tumor medially to the stylomandibular plane (tunnel) confirmed by MRI or CECT—68 and 46 cases, respectively. 

All calculations have been carried out by means of Microsoft Excel 2019 spreadsheet and STATISTICA, TIBCO Software Inc. (2020) Data Science Workbench (StatSoft Polska Sp. z o.o., Kraków, Poland), version 14. In the statistical description of quantitative data, classical measures of location such as arithmetic means and median and of variation such as standard deviation and range were used. The normality of distribution of the variables was tested by the use of the Shapiro–Wilk’s test. In order to compare groups in pairs for ordinal variables, the Mann–Whitney and Wilcoxon tests were used. The Kruskal–Wallis test was implemented to assess the differences in ordinal data among three or more independently sampled groups, and when the outcome was statistically significant, a multiple-comparisons post-hoc test (Dunn’s test) was subsequently used. Qualitative data were compared according to the number of cases in each compared category and/or their expected values; the Pearson’s chi-square test, Yates’ correction, or Fisher’s exact test were used. Univariate analyses by means of logistic regressions were carried out in order to evaluate the factors associated subsequently and independently with each of the considered dependent variables. The analyses included the determination of ORs, their respective 95% Cis, and significance levels. Subsequently, the multivariate logistic regression analyses were carried out, including all variables that presented statistically significant in the respective univariate analyses (either performed by comparisons of groups or univariate logistic regressions). In all the calculations the statistical significance level was set to *p* < 0.05.

## 3. Results

**Figure 2:**
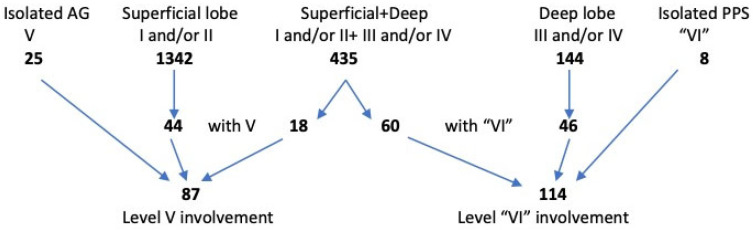
Location of 1954 primary parotid tumors according to ESGS classification (AG—accessory gland; PPS—parapharyngeal space).

Figure 2 and Table 1 show the exact location of tumors according to the ESGS levels; the PPS is included as level “VI”. Tumors of the parotid gland were, by far, most often detected in level II (83%) and level I (50%). Less frequently, the deep lobe was involved (levels III and IV, 26% and 9%, respectively). Tumors of the accessory gland (level V) were detected in only 4.5% of patients. In the analyzed group of patients, age ranged from 13 to 97 years, with a median age of 64 years. There was a slight predominance of women (53.8%). Non-malignant neoplasms predominated (93.37%). Pleomorphic adenomas (PAs) and Warthin tumors (WTs), 42.93% and 42.47%, respectively, were the most common histological diagnoses.

The involvement of the parapharyngeal space was found in 114 patients (5.83%). In the majority of cases, tumors involving the deep lobe or the entire gland were detected (in 46 and 60 cases, respectively). A PPS tumor originating from the protuberance of the deep lobe was found in only eight patients. Most PPS involvement was associated with tumor location in levels II and III (33), III (22), and III and IV (19), with other levels involved far less frequently (Table 1). Table 2 and Table 3 show the clinical and histological data as well as the extent of the treatment (according to the ESGS) of the entire analyzed group of patients.

All tumors were treated surgically with a transparotid approach (parotidectomy) in combination with transoral access in 43 patients, except for eight cases in which only transoral access was used (lesion limited to the PPS). Indication for combined surgery was a large (>4 cm) internal component of a dumbbell tumor. The transoral approach was limited to benign tumors, but combined transparotid/transoral was used either for benign or for malignant tumors. The PPS involvement of deep lobe/entire parotid gland tumors resulted in the necessity of subtotal or total parotidectomy, and in eight cases of malignant tumors, also in resection of the VII nerve with intentional paralysis (Table 3). All patients with malignant tumors received postoperative radiation therapy. Transient total or partial facial nerve dysfunction occurred in 40 cases (44.4%). Data about indefinite complications (hematoma and infection) were available from 76 patients, among them 8 had unproper wound healing. 

Follow-up occurred for 24 to 83 (average 50.4) months and full data were available in 102 patients. In 8 out of 84 patients with benign tumors revision surgery was needed because of residual disease, and full recovery was achieved in all but one patient (1.2%). In the group with malignant tumors, 8 of 18 (44.4%) patients died of the disease.

In a univariate analysis of tumors with and without involvement of the parapharyngeal space, statistically significant differences were found in the number of tumors >4 cm (12.97% vs. 37.72%), percentage of malignant tumors (7.12% vs. 17.55%), incidence of Warthin Tumors (WTs) (43.58% vs. 24.56%), percentage of R1 resection (5.53% vs. 12.50%), and rate of postoperative facial nerve paresis (17.15% vs. 53.34%). There were no significant differences between the two groups in demographics or FNAC result (Table 4). 

Multivariate analysis showed that tumors with parapharyngeal space involvement were statistically significantly characterized by larger size (tumors > 4 cm were 2.9 times more frequent), 2 times less frequent occurrence of WTs, and 1.6 times higher risk of postoperative VII nerve paresis (Table 5).

## 4. Discussion

Quer, in response to a letter to the editor regarding the necessity of addition of a new level to the ESGS classification, stated that “the resection of levels III–IV theoretically includes any parapharyngeal extension of the parotid gland, so I do not see a resection of levels III-IV preserving the parapharyngeal extension” [7]. The authors of this study cannot agree with this statement, since the extent of resection of levels I–V in PPS involvement varies depending on the tumor starting point (level), size, histology, and preferences of the operating surgeon, as depicted in the data shown in Table 4. Multiple techniques are used to obtain surgical access to deep lobe tumors with PPS involvement, and transparotid-transcervical approaches are the most frequently performed. However, it is possible to resect a PPS tumor (isolated but in continuity with the deep parotid lobe) from a transoral access, without removing levels III and/or IV. Transoral-only access is deba but can be considered on par with extracapsular dissection (ECD) of tumors of other levels, especially with endoscope-assisted or transoral robotic surgery (TORS). In cases of involvement of the prestyloid portion of the PPS by dumbbell parotid tumors, dual access (transparotid with transoral) is common, and for extremely large or recurrent tumors, as well as carcinomas, sometimes a wide transparotid–transcervical access in combination with mandibulotomy is necessary [3,8,9,10,11,12,13,14,15,16].

There is no consensus in the literature regarding the criteria for PPS involvement. According to Li et al. PPS involvement can be stated radiologically when the tumor is located medial to the stylomandibular plane and intraoperatively when it enters the PPS through a corridor bounded by the mandible, styloid process, and the stylomandibular ligament (the so-called stylomandibular tunnel, the narrowest point for the protuberance of the deep lobe). The aforementioned authors further divided PPS tumor extension into major and minor (>50% and ≤50% of cross-sectional area medial to the stylomandibular plane, respectively) [4]. Crossing of the stylomandibular plane appears to be an appropriate anatomic landmark, and a suitable criterion for PPS invasion by tumors of the deep lobe or the entire parotid gland. It is also worth emphasizing that in case of PPS involvement it is always necessary to perform diagnostic imaging based on MRI, and in the case of contraindications for this examination, CT with CE [2,8,10]. Imaging studies not only allow for the assessment of tumor size and location in the PPS but are also useful in differentiating between benign and malignant tumors [17].

As our results showed, there are statistically significant differences between parotid gland tumors with and without PPS involvement. Among the most important differences are the higher percentage of postoperative paresis of the VII nerve, the size of the tumors, and the ratio between PAs and WTs. 

The higher risk of VII nerve paresis can be easily explained by the necessity of displacing its trunk or branches to access a tumor located medially from it in the so-called deep lobe and/or the PPS [2,18]. In the material presented here, tumors > 4 cm were nearly three times more common in cases involving the PPS, which is consistent with the results of other authors [18,19,20]. In a study comparing PAs developing in the parotid gland and isolated in the PPS, tumors in the PPS were nearly two times larger. The authors explain this by indicating anatomical conditions favoring the development of larger tumors and their later clinical manifestation [20]. The most common clinical manifestation is an intraoral mass in combination with a parotid tumor, which immediately suggests a ‘dumbbell’ shaped deep lobe parotid tumor. A pathological mass without ulceration located exclusively in the pharynx (without parotid tumor) requires imaging studies to differentiate between the starting point in the pre- and post-styloid compartment of the PPS [1,8].

Hornung et al. also showed that pleomorphic adenomas of the PPS were more likely to have satellite nodules and a lower presence of an intact anatomical capsule [20]. The lack of ‘cuffing’ of the salivary gland parenchyma on the side of the PPS, and thus the need for intracapsular dissection (ICD, enucleation) instead of ECD, with a higher risk of capsule damage and more frequent satellite nodules, may explain the twofold higher rate of R1 resection of tumors with PPS involvement in our material (statistical significance in univariate analysis only). 

Lack of radicality during primary treatment may result in difficulties in disease control—as Polat et al. reported in their material, there are enormous difficulties in achieving complete resection during revision surgery for recurrent/residual PPS pleomorphic adenomas [21]. Different results were obtained by Mendelsohn et al., who found no association between positive surgical margins and risk of recurrence [22]. The vast majority of neoplasms developing in the parotid gland are benign in nature; however, in the material presented here, a 2.5 times higher incidence of malignant neoplasms was observed with PPS involvement. A disturbance of normal rate between malignant and benign tumors within parotid gland can be explained by lower occurrence of WTs in cases with PPS extension. As in the literature, the most common parotid gland tumor with PPS involvement was a PA; simultaneously, WTs were found to be much less frequent in the group with PPS involvement [2,19]. In our material, more than 13% of tumors spread to the PPS, which is slightly higher than the 10.3% presented by Li et al [4]. In a previous study, the involvement of the PPS by parotid carcinomas was found to be an outstanding adverse prognostic factor; however, statistical significance was demonstrated only in univariate analysis [23]. Li et al. found no statistically significant effect of PPS involvement on treatment outcome (margin status, recurrence, and survival) [4]. Histologically, malignant tumors of the parotid gland with PPS involvement are heterogeneous, without a predominance of a specific type of cancer [1,4,19]. It is worth highlighting the low sensitivity of FNAC in parotid tumors. In our material, about 29% of the results were negative or non-diagnostic in both tumors with and without PPS involvement, which is consistent with the results of other authors [2,19]. Some authors believe that there is no need to perform FNAC in PPS tumors, except for cases where a malignant process located in the prestyloid compartment is suspected, since the tumor most often originates in the parotid gland [18,24].

Our study has several limitations. First, the retrospective nature of the study and that only selected data collected in the database were available for analysis. Moreover, the diversity of histological types of neoplasms with a predominance of pleomorphic adenomas and Warthin tumors could have an influence on our results. Additionally, the limited follow-up (24–83 months) could affect the number of observed recurrences.

## 5. Conclusions

There are clear anatomical criteria for PPS involvement in parotid gland neoplasms. These neoplasms show some clinical and histological differences and require more complex surgical accesses. Therefore, they cannot be treated as “ordinary” tumors occupying the deep lobe or the even whole gland.

## Figures and Tables

**Table 1:** Involvement of ESGS levels (with additional level VI for PPS).

No (%) of Involved Levels						No (%) of pts
975 (49.89%)	1633 (83.57%)	514 (26.3%)	174 (8.9%)	87 (4.45%)	114 (5.83%)	
I	II					699 (35.77%)
	II					514 (26.3%)
	II	III				229 (11.71%)
I						85 (4.35%)
		III				57(2.91%)
I	II	III				50 (2.55%)
		III	IV			33 (1.68%)
	II	III			VI	33 (1.68%)
I			IV			30 (1.53%)
I	II	III	IV			28 (1.43%)
				V		25 (1.27%)
		III			VI	22 (1.12%)
		III	IV		VI	19 (0.97%)
I	II			V		16 (0.81%)
I				V		16 (0.81%)
	II			V		12 (0.61%)
	II	III	IV			11 (0.56%)
I	II	III	IV		VI	10 (0.51%)
I	II		IV			9 (0.46%)
I	II	III			VI	8 (0.4%)
I			IV		VI	8 (0.4%)
			IV			8 (0.4%)
					VI	8 (0.4%)
I	II	III	IV	V		7 (0.35%)
			IV		VI	5 (0.25%)
I			IV	V		5 (0.25%)
I	II	III		V		4 (0.2%)
	II	III		V		2 (0.1%)
	II	III	IV		VI	1 (0.05%)
						1954 (100%)

PPS—Parapharyngeal space.

**Table 2:** Clinical and histological features of the material.

Feature	*n*	%
Age		
Min–Max years	13–97	
Average (Median)	61 (64)	
Sex		
Male	902	46.2%
Female	1052	53.8%
Place of residence	(1895)	
Village	501	26.43%
Town	671	35.40%
City	723	38.15%
Tumors size	(1948)	
<2 cm	569	29.20%
2–4 cm	1098	56.36%
>4 cm	281	14.42%
Preoperative facial nerve palsy	53	2.71%
benign	2	0.11%
malignant	51	33.77%
Preoperative FNAB	1259	64.43%
Positive	902	71.64%
Negative (of malignant)	331 (46)	26.29% (45.54%)
Non diagnostic (of malignant)	26 (2)	2.06% (1.98%)
Kind of surgery		
Extracapsular dissection	114	5.83%
Partial superficial parotidectomy	334	17.09%
Lateral conservative parotidectomy	832	42.57%
Subtotal conservative parotidectomy	299	15.30%
Total conservative parotidectomy	328	16.78%
Total semiconservative parotidectomy	9	0.46%
Total radical parotidectomy	38	1.9%
Postoperative facial nerve status	(1654)	
Normal function	1374	83.07%
Paresis/palsy (unintentional)	280	16.92%
Total (all branches/trunk)	89	5.38%
Partial (branch/es)	191	11.54%
Single marg. mandibular branch	131	7.92%
Histology (WHO 2017)	(1954)	
Benign tumors	1803	93.37%
Malignant tumors	151	7.72%
Radicality (total/malignant)	(1633/116)	
R0	1513/82	92.65% (70.68%)
R1	96/28	5.87% (24.13%)
R uncertain	24/6	1.46% (5.17%)

**Table 3:** Extent of surgery according to the ESGS.

Kind of Surgery	ESGS	*n*	%
Extracapsular dissection (ECD)	ECD I	10	0.51
	ECD II	81	4.14
	ECD V	15	0.76
	ECD VI	8	0.40
Partial superficial parotidectomy (PSP) with accessory gland	PAR I	25	1.27
	PAR II	298	15.25
	PAR I, V	7	0.35
	PAR II, V	4	0.20
Lateral conservative parotidectomy (LCP)	PAR I, II	796	40.73
with accessory gland	PAR I, II, V	36	1.84
Subtotal conservative parotidectomy (STCP)	PAR I, II, III	134	6.85
	PAR I, II, IV	8	0.40
	PAR II, III	114	5.83
	PAR II, III, IV	11	0.56
with accessory gland	PAR I, II, III, V	2	0.10
	PAR I, II, IV, V	2	0.10
with parapharyngeal space	PAR I, II, III, VI	12	0.61
	PAR II, III, VI	16	0.81
Total conservative parotidectomy (TCP)	PAR I, II, III, IV	241	12.33
with accessory gland	PAR I, II, III, IV, V	17	0.87
with parapharyngeal space	PAR I, II, III, IV, VI	70	3.58
Total semiconservative parotidectomy (TSCP) *	PAR I, II, III, IV (VII) **	9	0.46
Total radical parotidectomy (TRP)	PAR I, II, III, IV (VII) ***	26	1.33
with accessory gland	PAR I, II, III, IV, V (VII)	4	0.20
with parapharyngeal space	PAR I, II, III, IV, VI (VII)	8	0.40

* some 7th nerve branches, ** with MM-7, S-6, *** with EAC-5, MM-4, TMJ-1, S-1. VI indicates Parapharyngeal space (PPS). PAR—parotidectomy; MM—masseter muscle; EAC—external auditory canal; S—skin; TMJ—temporomandibular joint.

**Table 4:** Comparative analysis of tumors with and without PPS involvement (univariate analysis).

Feature	No PPS Involvement	PPS Involvement	*p* Values *
Age (median)			
<64 years	923 (50.16%)	62 (54.86%)	0.331
>64 years	917 (49.84%)	51 (45.145)	
Sex			
Female	985 (53.54%)	67 (58.77%)	0.276
Male	855 (46.46%)	47 (41.23%)	
Place of residence			
Village	475 (26.40%)	26 (27.08%)	0.126
Town	629 (34.96%)	42 (43.76%)	
City	695 (38.64%)	28 (29.16%)	
Tumor size			
<2 cm	555 (30.26%)	14 (12.28%)	**0.000**
2–4 cm	1041 (56.77%)	57 (50.00%)	
>4 cm	238 (12.97%)	43 (37.72%)	
Malignancy			
No	1709 (92.88%)	94 (82.45%)	**0.000**
Yes	131 (7.12%)	20 (17.55%)	
FNAC			
Positive	858 (71.67%)	44 (70.96%)	0.966
Negative/non-diagnostic	338 (28.23%)	18 (29.04%)	
Histology			
Warthin’s tumor	802 (43.58%)	28 (24.56%)	**0.000**
Pleomorphic adenoma	784 (42.61%)	55 (48.25%)	
Other	254 (13.81%)	31 (27.19%)	
Radicality of resection			
R0	1431 (93.11%)	82 (85.42%)	**0.015**
R1	85 (5.53%)	12 (12.50%)	
R uncertain	21 (1.36%)	2 (2.08%)	
Postoperative 7th nerve palsy			
No	1328 (82.85%)	42 (46.66%)	**0.000**
Yes	275 (17.15%)	48 (53.34%)	

FNAC—fine needle aspiration cytology. * *p* values in bold: statistically significant.

**Table 5:** Multivariate analysis. Clinical and pathological features that differentiate tumors with and without PPS involvement.

Feature	Estimate	Odds Ratio	95% CI	*p* Values *
Tumor size > 4 cm	1.064	2.899	1.989–4.226	**0.000**
Malignancy risk	−0.839	0.432	0.181–1.033	0.059
No WT histology	0.695	2.003	1.359–2.954	**0.000**
R1 radicality of resection	0.099	1.104	0.874–1.395	0.405
PO 7th nerve palsy risk	0.474	1.607	1.357–1.902	**0.000**

PO—postoperative. * *p* values in bold: statistically significant.

## Data Availability

The data is available upon request with limitations due to anonymity and ethical considerations.
